# From Pap Test to Treatment: Assessing Facility Level Gaps in Cervical Cancer Care in Cape Town

**DOI:** 10.1002/puh2.70360

**Published:** 2026-09-04

**Authors:** Greta Sophie Neethling, Rubina Razack, Matthys Hendrik (Hennie) Botha

**Affiliations:** ^1^ National Health Laboratory Service and Department of Pathology Division of Anatomical Pathology Faculty of Medicine and Health Sciences Stellenbosch University Cape Town South Africa; ^2^ Unit Gynaecological Oncology Department of Obstetrics and Gynaecology Faculty of Medicine and Health Sciences Stellenbosch University and Tygerberg Hospital Cape Town South Africa

**Keywords:** cervical disease, colposcopy clinic attendance rate, continuity‑of‑care

## Abstract

**Introduction:**

The World Health Organization (WHO) 90‑70‑90 cervical cancer elimination targets aim for 90% human papillomavirus (HPV) vaccination rates, 70% cervical screening rates, and 90% treatment rates for women with cervical disease. Although progress has been made in vaccination and screening in South Africa, less is known about treatment coverage following abnormal screening results. We focused on adherence to colposcopy clinic referrals among women with abnormal cytology.

**Methods:**

We conducted a retrospective descriptive study of cervical cytology samples collected in 2022 from women attending 19 public healthcare facilities within a sub‑district of Cape Town. All cytology results reported as high‑grade intraepithelial lesions or invasive cervical carcinoma (HSIL+) were included. Colposcopy clinic attendance was defined as documented histological sampling within 24 months of diagnosis. Colposcopy clinic attendance rates (CCARs), test time intervals, and distance from referral hospital were calculated for each facility. Facilities were compared using percentile distributions, and distance to referral hospitals was analyzed as a potential spatial barrier.

**Results:**

Of 9,069 cervical samples, 556 (6.1%) were reported as HSIL+. Overall, 65.8% of women underwent histological sampling within 24 months. CCARs varied across facilities, ranging from 42.5% to 87.5% (mean 63.1%). Distance to referral hospitals (range 0–22 km; mean 10.8 km) showed no correlation with colposcopy clinic attendance.

**Conclusion:**

A substantial gap exists between current CCARs and the WHO treatment target. Variability among facilities, independent of geographic distance, suggests that health system factors, such as communication, recall, and referral processes, play a key role in treatment outcomes.

## Graphical abstract

Colposcopy clinic attendance in women with cervical precancer remains below the WHO treatment target. As distance to referral hospitals was not associated with follow‐up outcomes, other barriers contributing to the care gap should be explored.

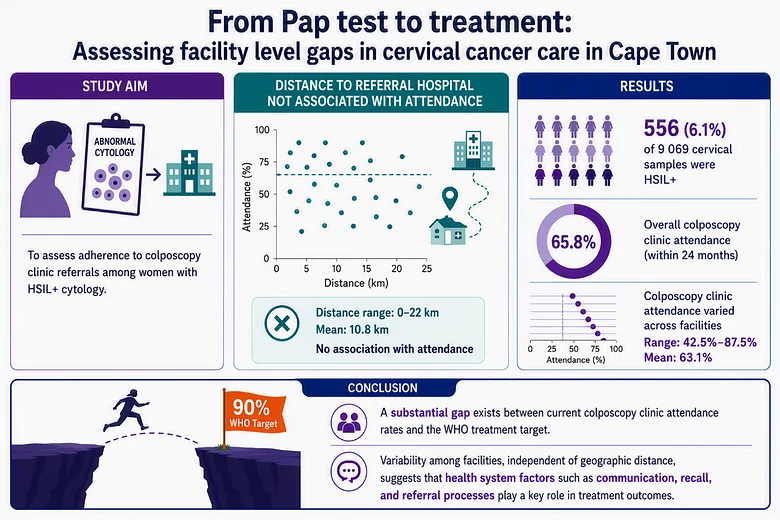

## 1 Introduction

The World Health Organization (WHO) 90‑70‑90 Cervical Cancer Targets Include: [1]

- 90% of girls fully vaccinated with the human papillomavirus (HPV) vaccine by age 15,
- 70% of women screened with a high‑performance test at ages 35 and 45, and
- 90% of women identified with cervical disease receiving appropriate treatment.

Will low‑ and middle‑income countries (LMICs) be able to come close with limited healthcare infrastructure, a shortage of skilled healthcare workers, widespread poverty, and low levels of cervical cancer awareness? Under such conditions, achieving these targets within the next 5 years can seem unrealistic. Although numerous initiatives in South Africa focus on vaccination and screening, our study is focused on the 90% treatment target.

Cervical cancer is a largely preventable and treatable disease, primarily caused by persistent infection with high‐risk HPV [2]. Despite this, it remains a leading cause of cancer‐related mortality in LMICs, largely due to inadequate access to screening and treatment services. In South Africa, cervical cancer is the most common cancer among women aged 15–44 years, with substantial consequences for young families [3]. The WHO aims to prevent 74 million new cervical cancer cases and 62 million deaths within the next century with its 90‐70‐90 targets. The elimination threshold is defined as an incidence rate of fewer than 4 cases per 100,000 women per year [1]. Recent data suggests that the crude incidence rate amongst South African women is 35.6 per 100,000 women per year [4]. Rising cervical cancer rates in South Africa are fuelled by human immunodeficiency virus (HIV), with the country facing one of the highest HIV prevalences in the world [5, 6].

To better understand South Africa's cervical precancer treatment performance, we conducted a review of available literature incorporating nine studies that assessed treatment coverage among women diagnosed with precancerous and cancerous cervical lesions (Table 1). These studies represented data from four of the nine provinces and treatment coverage varied substantially across publications. Gauteng province contributed three studies, which showed the least variability but also the lowest treatment coverage (25.6%–27.8%) [7, 8, 9]. The Eastern Cape and KwaZulu‑Natal were each represented by a single study, reporting treatment coverage of 54% and 63.5%, respectively [10, 11]. The Western Cape contributed four studies, with treatment coverage ranging widely from 38.2% to 85.1% [12, 13, 14, 15]. Overall, the review revealed a significant gap between current treatment coverage and the WHO 90% target. These findings underscored the need for further investigation into health system barriers and led to our current project.

**TABLE 1:** Literature summary of treatment coverage in precancerous and cancerous lesions diagnosed using cytology in South Africa.

Reference	Location	Number of cases	% Colposcopy follow‐up
Jassat et al. [7]	Johannesburg Metro, Gauteng	557	27.8
Murie and De Sa [14]	Cape Town Metro, Western Cape	1886	51.0
Blanckenberg et al. [12]	Overstrand, Western Cape	103	51.3
Knegt [10]	Referred to East London, Eastern Cape	842	54.0
Katz et al. [11]	Durban, KwaZulu‐Natal	50	63.5
Guzha et al. [13]	Referred to Cape Town, Western Cape	4036	85.1
Rohner et al. [9]	Johannesburg, Gauteng	688	25.6
Neethling and Razack [15]	Referred to Parow, Western Cape	204	38.2
Juggernath et al. [8]	Johannesburg, Gauteng	4182	26.6

The aim of this study is to measure the colposcopy clinic attendance rate (CCAR) among women with Pap test results of high‐grade intraepithelial lesions or invasive cervical carcinoma (HSIL+). Our primary focus was to assess loss to follow‐up. Therefore, the outcome of interest was adherence to the referral pathway from cervical screening to colposcopy clinic attendance, rather than the completion or success of cervical disease treatment.

We also sought to identify clinics with high colposcopy clinic referral adherence among patients to compare their continuity‐of‐care strategies with those of lower performing clinics. Distance between primary healthcare facilities and referral hospitals may be a critical spatial barrier that influences access to treatment. Because participants must pay for their own transport, financial constraints in poorer communities may reduce their ability to access services at referral hospitals. Longer distances have been shown to negatively affect attendance, particularly for follow‐up appointments [16].

Anecdotal evidence suggests that primary healthcare facilities in this sub‐district lack standardized communication, recall, and referral processes. To meaningfully compare strategies across facilities, the first step is to measure the proportion of women with HSIL+ who attended their colposcopy clinic visit from each facility.

## 2 Methods

We conducted a retrospective descriptive study using cervical cytology samples (liquid‐based and conventional) collected in 2022 from women attending 19 public primary healthcare facilities in a sub‐district of Cape Town, South Africa. These samples were obtained as part of routine national cervical cancer screening services. All cytology results interpreted as HSIL+ were included. These included high‐grade intraepithelial lesions or invasive cervical carcinoma of squamous or endocervical glandular origin.

Data were obtained from the National Health Laboratory Service (NHLS) Corporate Data Warehouse (CDW), a centralized repository of all public sector laboratory results in South Africa. The dataset included patients’ age, primary healthcare facility attended, date of cytology test report, cytology diagnosis, date of histology sample taken, histology diagnosis, histological test type, and HIV status. Data were received and maintained in a Microsoft 365 Excel spreadsheet (.xlsx). At least 2 years were allowed after the initial cytology report to ensure adequate opportunity for treatment.

Cytology, histology, and HIV records were matched using the unique patient identifier (UPI) provided by the CDW. Patient identifiers were withheld for confidentiality reasons, and therefore all record linkage and analyses were performed using the UPI. A UPI is a distinct number assigned to a single individual across South Africa's health system, ensuring medical records and test results are accurately linked. The patient's name, surname, date of birth, gender, national registration number and other necessary personal details are used when creating the UPI.

Duplicate cases (i.e., same UPI) were merged into a single record where more than one cytology or histology tests were performed. Missing HIV status data were supplemented using available viral load, CD4 count or HIV test results as proxy measures of HIV status. When a histology report was absent, it was accepted that the patient did not have histological sampling. Histological sampling was recorded as (i) excision, (ii) biopsy only, or (iii) biopsy with subsequent excision, hysterectomy, or chemo/radiation treatment.

CCARs and time intervals between cytology and histology tests were calculated for each facility. Colposcopy clinic attendance refers to a patient's visit at the referral hospital for evaluation and histological sampling following an abnormal cervical cytology result, representing the first step in treatment. As we only had the UPI, we were unable to confirm colposcopy clinic attendance on the provincial electronic health records and therefore used histological sampling as proxy for attendance.

$$\begin{array}{c} \mathrm{CCAR}\left(\%\right)=\left[\frac{\mathrm{Number}\;\mathrm{of}\;\mathrm{women}\;\mathrm{who}\;\mathrm{had}\;\mathrm{histological}\;\mathrm{sampling}\;\mathrm{during}\;\mathrm{colp}\;\mathrm{visit}}{\mathrm{Total}\;\mathrm{number}\;\mathrm{of}\;\mathrm{women}\;\mathrm{requiring}\;\mathrm{cervical}\;\mathrm{treatment}}\right] \end{array}$$

Mean values, standard deviations, and percentile distributions were used to identify clinics below the 25th percentile and above the 75th percentile using IBM SPSS Statistics version 31. In addition, distance between primary healthcare facilities and referral hospitals was calculated using Google Maps and analyzed as an independent variable in relation to CCARs.

Ethical approval was granted by the Stellenbosch University Health Research Ethics Committee (S25/02/014), with additional approvals from the NHLS, the Provincial Department of Health, and the City of Cape Town. Waiver of patient consent was approved since retrospective, routinely reported laboratory data was used.

## 3 Results

In 2022, a total of 9069 cervical samples were received from the 19 primary healthcare facilities. HSIL+ was reported in 556 women (6.1%), for whom colposcopy referral was recommended. Of these results, 95.5% were high‐grade squamous intraepithelial lesion (*n* = 531), 0.2% were adenocarcinoma‐in‐situ (*n* = 1), and 4.3% were carcinoma (*n* = 24). The mean age of women with HSIL+ was 39.4 years (range 16–82). HIV status was positive in 77.2% (*n* = 429), negative in 18.3% (*n* = 102), and unknown in 4.5% (*n* = 25) of women. Of the 556 women with HSIL+, 366 (65.8%) underwent histological sampling within 24 months of cytology diagnosis. Excision (large loop excision of the transformation zone or cone biopsy) was obtained in 320 women (87.4%), a single diagnostic biopsy in 30 women (8.2%) and 16 women (4.4%) had a diagnostic biopsy with subsequent excision, hysterectomy, or chemo/radiation treatment.

**FIGURE 1:**
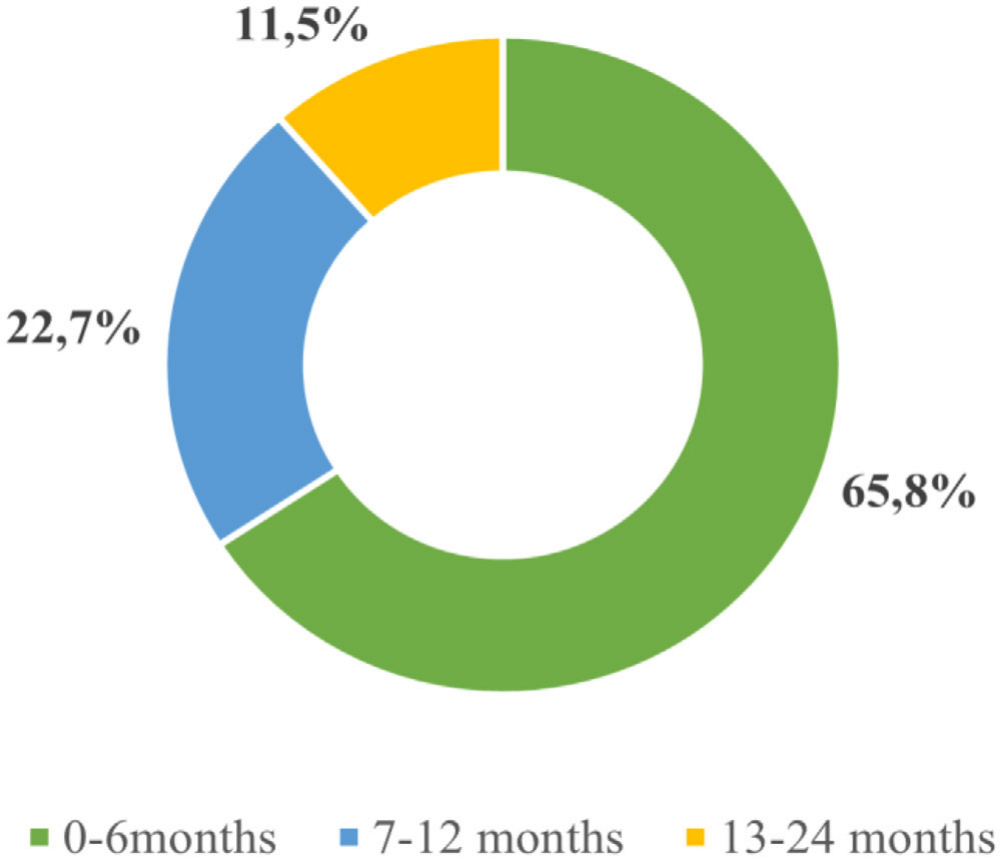
Interval between cytology and histology samples.

Most histological samples were obtained within the first 6 months (65.8%; *n* = 241), with an additional 22.7% (*n* = 83) between 7 and 12 months and 11.5% (*n* = 42) between 13 and 24 months. A total of 190 women (34.2%) had no histology submitted to the NHLS within 24 months after initial cervical cytology sample (Figure 1).

CCARs varied substantially across facilities, ranging from 42.5% to 87.5%, with a mean of 63.1%; 95% CI (56.2, 70.0); and a standard deviation of 14.4 (Table 2).

**TABLE 2:** Summary of cytology and follow‐up during 2022 from the 19 facilities in the sub‐district.

	Clinic ID	Distance from hospital (km)	Total samples submitted	HSIL+ lesions on cytology	HSIL+ proportion (%)	HSIL+ lesions sampled	CCAR (%)
Above 75th percentile	1	11	486	24	4.9	21	87.5
	2	5.8	733	49	6.7	42	85.7
	3	0	452	21	4.6	18	85.7
	4	22	757	28	3.7	21	75.0
	5	8	781	70	9.0	51	72.9
Mid 50 percentile	6	11	435	25	5.7	18	72.0
	7	19	924	43	4.7	30	70.0
	8	6.3	867	73	8.4	47	64.4
	9	13	462	25	5.4	16	64.0
	10	15	294	25	8.5	16	64.0
	11	6.4	288	13	4.5	8	61.5
	12	14	231	13	5.6	8	61.5
	13	16	601	42	7.0	23	54.8
Below 25th percentile	14	1.9	150	6	4.0	3	50.0
	15	8.4	221	10	4.5	5	50.0
	16	6.4	61	4	6.6	2	50.0
	17	21	530	38	7.2	17	44.7
	18	11	144	7	4.9	3	42.9
	19	8.9	652	40	6.1	17	42.5
Total		**10.8 (mean)**	**9069**	**556**	**6.1 (mean)**	**366**	**65.8 (mean)**

Abbreviation: CCAR, colposcopy clinic attendance rate.

**FIGURE 2:**
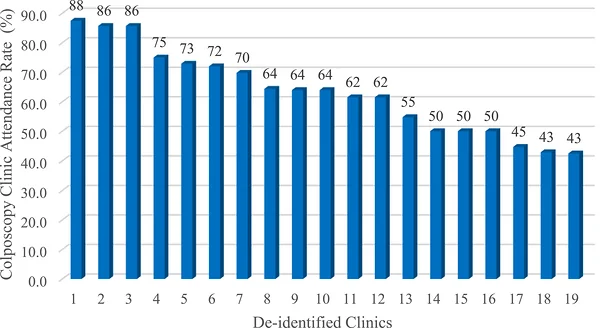
Colposcopy clinic attendance in women from primary health care facilities in the same sub‐district.

A total of six facilities were classified in the first quartile, with CCARs up to 50.0%, whereas five facilities were grouped in the fourth quartile, with attendance rates starting at 72.9% (Figure 2).

Distance was evaluated as a spatial barrier, with travel distances to referral hospitals ranging from 0 to 22 km, mean of 10.8 km, 95% CI [7.9, 13.7], and standard deviation 6.0 (Table 3). The average distance travelled was divided into three categories: upper 75th percentile, mid 50, and lower 25th percentile (Table 3).

**TABLE 3:** Primary health care facility colposcopy clinic attendance rates compared to distance from referral hospital.

Facilities	Range (km)	Mean distance (km)	95% CI	Standard deviation
Above 75th percentile	0–22	9.4	−0.7, 19.5	8.1
Mid 50 percentile	6.3–19	12.6	8.8, 16.3	4.5
Below 25th percentile	1.9–21	9.6	2.9, 16.3	6.4
Total	**0–22**	**10.8**	**7.9, 13.7**	**6.0**

**FIGURE 3:**
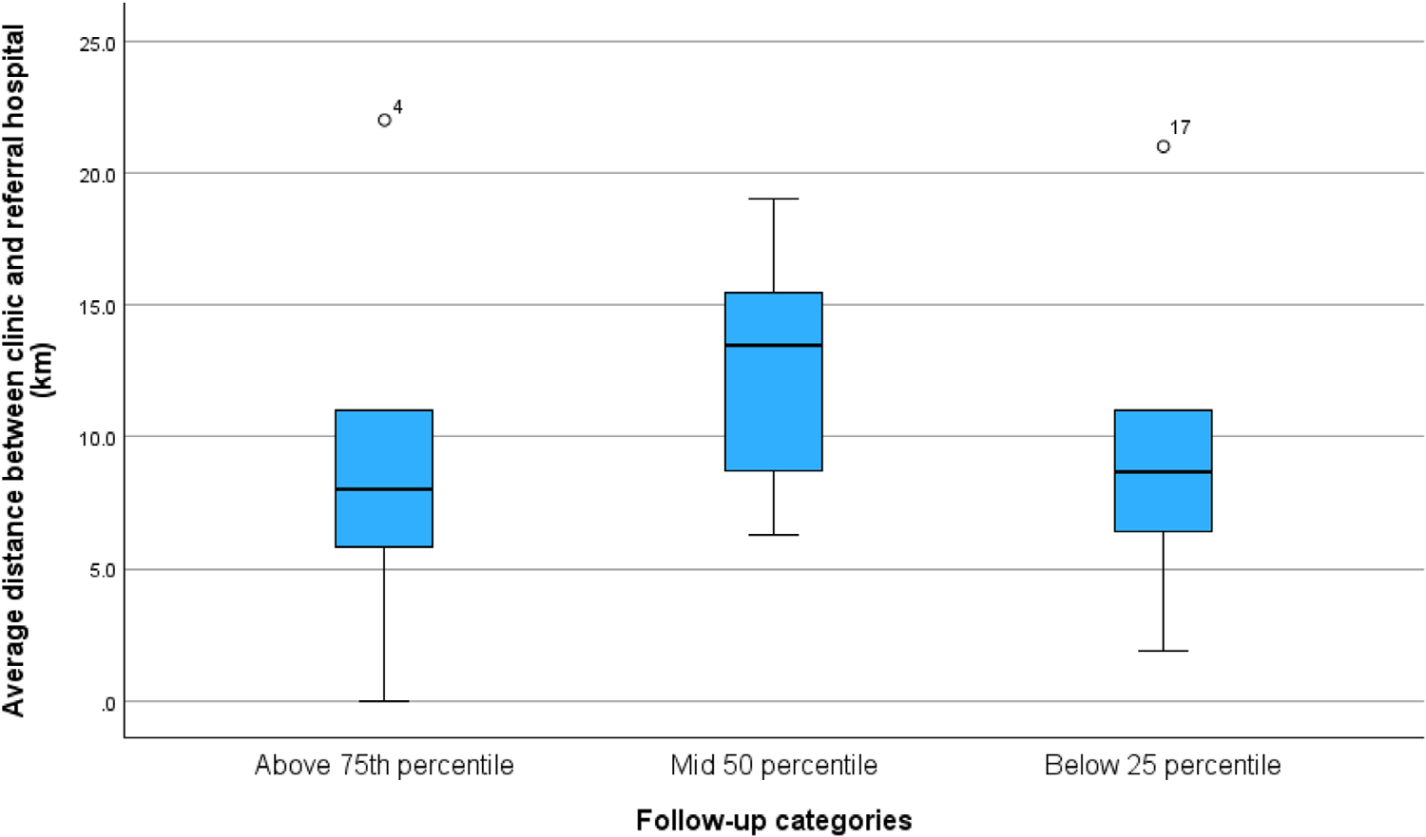
Correlation between distance from primary healthcare facility to referral hospital.

Distance was not significantly associated with CCARs in this dataset (Figure 3). Pearson's correlation coefficient indicated very weak negative relationships (*r* = −0.11, *p* = 0.64). From Figure 3 it is apparent that facilities from opposite ends of the spectrum had similar distance points and means.

## 4 Discussion

The primary objective of any cervical cancer screening program is to prevent cervical cancer. Achieving this requires a comprehensive approach that includes HPV vaccination, effective screening, and the timely treatment of cervical lesions following an abnormal screening result. This study supports that goal by measuring CCARs among women with Pap test results of HSIL+ in a sub‑district of Cape Town.

This study found substantial variation in CCARs across treatment facilities (42.5%–87.5%). Three facilities performed particularly well, achieving treatment rates above 85% and nearly reaching the WHO treatment target. The current finding of 42.5%–87.5% variation in attendance rates across facilities in Cape Town is consistent with the literature, that is, 25.6%–85.1% variation in treatment coverage across South Africa [7, 8, 9, 10, 11, 12, 13, 14, 15].

Nearly two thirds of women who were seen at a referral hospital in Cape Town received histological sampling within 6 months of receiving a Pap test result, with 86% receiving sampling within 1 year. These results suggest that colposcopy clinic waiting times and service capacity in this area are generally acceptable. Distances between primary healthcare facilities and referral hospitals were not associated with CCARs. Facilities in the above 75th percentile and below the 25th percentile groups had the same distance range and mean. Pearson's correlation coefficient indicated very weak negative relationships, underscoring the need to explore other barriers contributing to variations in treatment coverage observed.

A limitation of this study was that patient identifiers were unavailable to the research team for confidentiality reasons, restricting analyses to the UPI provided by the CDW. Consequently, colposcopy clinic attendance could not be directly verified using provincial electronic health records, and documented histological sampling was used as a proxy for attendance. Although some women may attend a colposcopy clinic without undergoing histological sampling, such cases are expected to be rare and may include women who are pregnant at the time of assessment. Therefore, the potential underestimation of colposcopy clinic attendance is likely to be minimal.

Cervical screening is of limited value if it is not linked to effective treatment. Measurements of treatment coverage following abnormal cervical screening test results are essential for auditing the effectiveness of cervical screening programs. Future studies will require root cause analyses to discover barriers to treatment and lean management methods to formulate methods to overcome those barriers.

With most South African provinces transitioning to primary HPV screening during 2024 and 2025, effective continuity‐of‐care processes will remain critical, particularly given that point of care testing and immediate treatment are not yet widely available. The faster turnaround time of HPV results compared with cytology may reduce loss to follow‐up, although further research is needed to confirm this potential benefit.

## 5 Conclusion

Despite timely attendance among women who accessed colposcopy services, histological sampling rates following HSIL+ on cytology remained substantially below the WHO 90% target in this Cape Town sub‑district. Marked variation in CCARs between primary healthcare facilities, independent of distance to referral hospitals, suggests that health system factors rather than geographic barriers are the primary determinants of treatment. Differences in communication of results, recall mechanisms, and referral processes are likely to contribute to this variability and should be further investigated.

These findings highlight the importance of strengthening continuity‑of‑care systems to ensure that women diagnosed with HSIL+ receive timely and appropriate treatment. Monitoring CCARs at facility level provides a valuable opportunity to identify high‑performing clinics and examine practices that may be transferable to lower‑performing settings. As South Africa transitions to primary HPV screening, robust follow‑up and referral pathways will remain essential to maximize the public health impact of cervical cancer screening programs. Screening alone is insufficient in the absence of effective treatment, and closing this gap is critical to achieving cervical cancer elimination goals.

## Author Contributions

Greta Sophie Neethling is the first and corresponding author (prepared the first draft) who planned and performed data collection and analysis. Greta Sophie Neethling also conducted project administration and funding acquisition. Rubina Razack and Matthys Hendrik (Hennie) Botha provided valuable guidance in conceptualization, ongoing support, and supervision. All authors reviewed and edited drafts of this manuscript and approved the final version of this article.

## Funding

This work was supported by The Global Cervical Cancer Prevention Project during study design and data analysis.

## Disclosure

The corresponding author is an early career researcher, and this work is part of her doctoral studies. The research findings and recommendations do not represent an official view of the City of Cape Town.

## Ethics Statement

Ethical approval was granted by the Stellenbosch University Health Research Ethics Committee (S25/02/014), with additional approvals from the NHLS, the Provincial Department of Health, and the City of Cape Town. The study was conducted in compliance with the principles outlined in the Declaration of Helsinki, the South African National Health Act (No. 61 of 2003), the Department of Health: Ethics in Health Research—Principles, Processes, and Structures (2015), and all relevant Stellenbosch University policies and guidelines.

## Consent

Waiver of consent has been approved.

## Conflicts of Interest

The authors declare no conflicts of interest.

## Statements Relating to Ethics and Integrity Policies

The author confirms compliance with academic integrity standards and declares no misconduct. Ethical approval was obtained from the relevant ethics review committee. No patient identifiers were used, and data were processed in concordance with the POPI Act.

## Data Availability

The data that support the findings of this study are available on request from the corresponding author. The data are not publicly available due to privacy or ethical restrictions.
